# Treatment of the Genito-Urinary Organs

**Published:** 1878-12

**Authors:** 


Treatment of the Genito-Urinary Organs, the use of Electricity, Damiana, etc., etc. By John J. Caldwell, M. D., Baltimore.

A Pamphlet of eight pages, on the above subject, with a number of cases illustrative of the beneficial effects of Damiana, alterative and tonic, and of electricity as a dynamic stimulant.

The paper is suggestive of thought as to the good effect of moral suasion in conjunction or precedent to medicinal remedies To persuade the victim of self-abuse or the roué to be a man, and that self-restraint is absolutely essential, is often the most difficult part of the discreet physician's task; and the proper treatment of the sexually diseased can never be overestimated. That they fall into the hands of the quacks and charlatans, who prey alike on their fears and purses, is a doom the honest physician should labor to save them from, and the works of Acton, Napheys, Storer, and others, in this direction, cannot be too highly valued. This contribution of Dr. Caldwell to the strictly professional literature of this subject is in the main favorable to the therapeutic action of Damiana, a rather new remedy in sexual disorders. H.

